# Multiresolution Analysis of HRRR Meteorological Parameters and GOES-R AOD for Hourly PM_2.5_ Prediction

**DOI:** 10.1021/acs.est.4c03795

**Published:** 2024-11-01

**Authors:** Dimple Pruthi, Qingyang Zhu, Wenhao Wang, Yang Liu

**Affiliations:** Gangarosa Department of Environmental Health, Rollins School of Public Health, Emory University, Atlanta, Georgia 30322, United States

**Keywords:** Wavelet transform, GOES-R, HRRR, Aerosol optical depth, PM_2.5_, Random forest

## Abstract

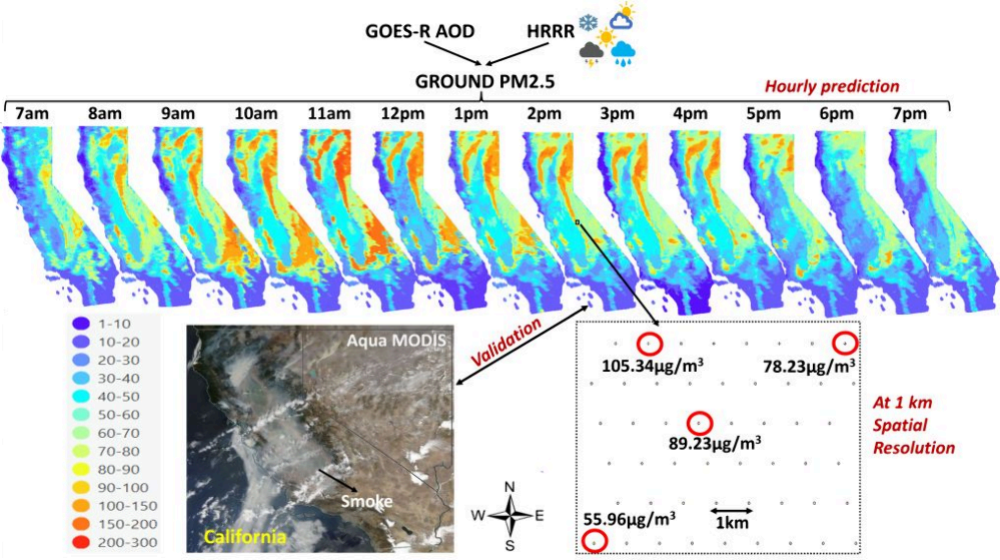

High-resolution, comprehensive exposure data are crucial for accurately estimating the human health impact of PM_2.5_. In recent years, satellite remote sensing data have been increasingly utilized in PM_2.5_ models to overcome the limited spatial coverage of ground monitoring stations. However, data gaps in satellite-retrieved parameters such as aerosol optical depth (AOD), the sparsity of regulatory air quality monitors for model training, and nonlinear relationships between PM_2.5_ and meteorological conditions can affect model performance and cause data gaps in most existing PM_2.5_ models. In this study, spatial gaps in AOD obtained from Geostationary Operational Environmental Satellite-16 are filled using Goddard Earth Observing System Composition Forecasting AOD estimations. Furthermore, to improve model performance, meteorological predictors such as temperature from the High-Resolution Rapid Refresh model are preprocessed using Daubechies wavelet to extract low and high frequency components. The spatially gap-filled AOD, along with meteorological data, are ingested into a machine learning model to predict hourly PM_2.5_ at a 1 km spatial resolution in California. The model evaluation metrics (OOB (out-of-bag) R^2^ = 0.86 and RMSE (root-mean-square error) = 9.27 μg/m^3^ and 10-fold spatial cross-validation R^2^ = 0.82 and RMSE = 9.82 μg/m^3^) demonstrate the model’s reliability in predicting ambient PM_2.5_, especially for states like California that experience frequent episodes of wildfires.

## Abstract 2

By incorporating the spatially filled GOES-AOD high resolution product and using wavelet transforms, the hourly seamless PM_2.5_ concentrations across California are well estimated at 1 km.

## 1 Introduction

Climate change leads to changes in temperature and precipitation patterns, resulting in an anticipated rise in both the frequency and intensity of wildfires across various regions globally. Exploring the intricate connections between meteorological factors and air pollution patterns is highourly significant for advancing the assessment of air pollution exposure and related epidemiologic investigations. Mounting evidence indicates the impacts of PM_2.5_ on various organ systems encompassing cardiovascular effects,^1,2^ respiratory implications,^3,4^ and consequences on obstetric outcomes,^5−7^ as well as effects on neural and cognitive abilities.^8,9^ A recent investigation revealed a 70% surge in out-of-hospital cardiac arrests associated to smoke exposure during severe wildfire events, particularly impacting individuals in lower socioeconomic groups.^10^ It is therefore crucial to monitor and evaluate PM_2.5_ levels during and following wildfires to effectively address the potential health and environmental repercussions linked to these events.

Over the course of several years, extensive efforts have been undertaken to predict ambient PM_2.5_ levels across different spatial and temporal scales, employing a range of ground, satellite, and CTM models-based data. As efforts and resources continue to grow over time, the objective is to achieve seamless ground PM_2.5_ data with a fine spatial and temporal resolution. However, some limitations exist in the existing PM_2.5_ prediction models. One of the major limitations is that the existing studies are often limited to small regions or short time scales. For example, Lee estimated annual average ambient PM_2.5_ concentrations at 1 km.^11^ Aguilera et al. and Di et al. estimated daily PM_2.5_ at the ZIP code level and 1 km, respectively.^12,13^ Li et al. developed a PM_2.5_ prediction model at a 1 km scale, with weekly temporal resolution.^14^ These studies employing advanced deep learning models still face challenges in achieving simultaneous fine spatiotemporal resolutions. A few works specifically address the PM_2.5_ modeling during major wildfire events.^15,16^ Some of the above-mentioned research has spatial gaps in PM_2.5_ prediction leading to biased prediction, missing a larger population unmonitored.^12,15^ In contrast to these approaches, our study aims to provide hourly PM_2.5_ levels without any spatial gaps at high spatial resolution of 1 km.

Additionally, many prediction models include ground air quality measurements as predictors. For example, in a PM_2.5_ prediction model developed by Li et al.,^14^ ground-level carbon monoxide measurements served as an important predictor. Hamed et al. and Zhang et al. used ground observations of other air pollutants (e.g., PM10) as the predictor for PM_2.5_.^17,18^ In these cases, the PM_2.5_ prediction model depends on the ground observation which has various limitations, e.g., nonuniform sensors distribution, high cost, etc. While the contribution of meteorological variables in estimating PM_2.5_ levels is well recognized,^19,20^ coarse-resolution meteorological parameters were often used. In addition, while most sophisticated prediction models allow nonlinear and nonparametric relationships between meteorological parameters and ground PM_2.5_ levels, no further processing of these parameters was performed. Such simple treatment ignores the multiresolution impact of meteorological conditions on PM_2.5_ production and transport at different scales and frequencies. For example, the short-term variation of temperature may affect PM_2.5_ formation and dispersion differently from longer-term trends of temperature. Additional decomposition of various meteorological parameters may allow for the identification of specific time-frequency components of weather fluctuations that are most strongly correlated to PM_2.5_ levels, thereby improving the accuracy of prediction models.

There has been significant advancement in machine learning models for PM_2.5_ prediction in recent years. While classical machine learning models (e.g., random forest, support vector machines, decision trees) continue to be employed, more complex models that require higher computational costs, such as convolutional neural networks (CNNs), recurrent neural networks (RNNs), and long short-term memory networks (LSTM), have also been utilized. CNNs integrated with other models like LSTM^21,22^ and random forests^23,24^ have also been reported in the literature. As the complexity of these newer model architectures increases, so does the computational cost. Leveraging cutting-edge GPUs provided by cloud services for deep learning can lead to significant training expenses without necessarily achieving major improvements in model performance.^25,26^ An alternative approach to achieving precise and efficient predictions is through refining the model inputs. Incorporating satellite-retrieved aerosol optical depth (AOD) as a predictor for PM_2.5_ is an example of this strategy.

In this study, we report our development of an hourly PM_2.5_ concentration model in California at 1 km spatial resolution using hourly AOD retrieved by geostationary satellites. Instead of employing a more complex machine learning algorithm to construct an efficient PM_2.5_ hourly prediction model, we took a different approach by performing wavelet transformation on the high-resolution meteorological data to identify significant features and abrupt spatial changes. Such an approach allows our model to capture the intricate dynamics of PM_2.5_ concentrations without relying on computationally expensive deep learning architectures.

## 2 Materials and Methods

### 2.1 Data Collection and Preprocessing

In this study, we employed GOES-R AOD, HRRR meteorological variables, and GEOS-CF AOD to predict hourly ambient PM_2.5_ measurements across California at 1 km. The input data undergo preprocessing involving calibration, interpolation techniques, and transformation. Details regarding the data sets and preprocessing techniques are provided in the subsequent sections.

#### 2.1.1 Study Area

**Figure 1:**
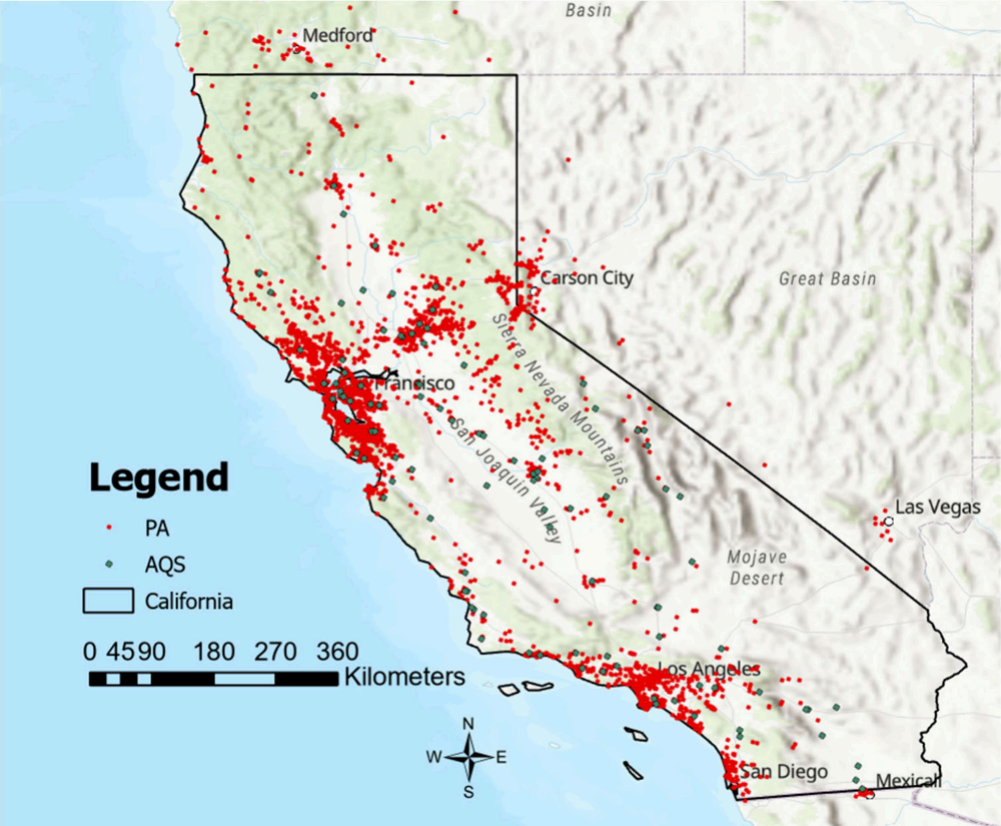
Study area, California. EPA AQS and PurpleAir monitors are marked in green and red, respectively.

California, positioned as the third-largest state in terms of both size and population, is home to 39 million residents and covers an expansive 423,970 km^2^ in the western USA, bordering the Pacific Ocean. Predicting the spatiotemporal distribution of PM_2.5_ across extensive and diverse regions like California, characterized by significant variations in emission sources, population, topography, meteorological conditions, and land-use, poses a considerable challenge. A 1 km modeling grid was created to achieve spatial consistency of all model parameters. The study area comprises a total of 493,561 grid cells (Figure 1).

#### 2.1.2 Ground PM_2.5_ Data

Hourly level PM_2.5_ measurements were obtained from the EPA’s Air Quality System (AQS) network, spanning the years 2019 to 2021 during the months of May to December (https://www.epa.gov/aqs). The fire season in California typically commences around July, coinciding with periods of heightened dryness and heat. To provide more accurate insights into model performance during wildfire episodes, particularly in predicting high PM_2.5_ levels, we have limited our analysis to specific time periods. Within California, a total of 104 stations supplied 762,112 hourly measurements throughout the study period.

Recent studies suggest that low-cost air quality sensors show potential in broadening the coverage of air quality information both temporally and spatially.^27,28^ These sensors can be easily deployed and relocated to various locations, as necessary. Nevertheless, numerous challenges such as precision, consistency, repeatability, and calibration of sensor measurements have been reported.^29^ In this study, we have incorporated PurpleAir (PA) measurements into model training. These measurements (https://www.purpleair.com/) were calibrated using geographically weighted regression (GWR).^28^ A total of 2980 sensors in California provided 9,265,016 hourly measurements throughout the study period. PM_2.5_ measurements from both AQS and PA monitors are allocated to nearest 1 km grid cells. Considering the AQS measurements as the gold standard, the measurements from PA were omitted in grid cells, where both AQS and PA data were present. Additionally, grid cells with multiple AQS or PA measurements underwent averaging to ensure a single PM_2.5_ measurement in a grid cell. It was noted that the training data exhibited only a limited number of instances with high-level PM_2.5_ concentrations. In this scenario, we employed the Synthetic Minority Oversampling Technique (SMOTE) to generate synthetic samples for instances with high PM_2.5_ levels. For all measurements in the minority class, the SMOTE function created synthetic observations along with their predictors by utilizing information from the five nearest neighbors.^15^

#### 2.1.3 Satellite Data

Launched in November 2016, GOES-16 operates in the eastern part of the United States at 75.2°W and is a geostationary weather satellite featuring the Advanced Baseline Imager (ABI) with 16 spectral bands in both visible and infrared wavelengths.^30^ ABI is designed to offer high-resolution imagery and radiometric data of the terrestrial surface, accompanied by atmospheric and cloud cover information.^31^ ABI’s AOD product has a temporal and spatial resolution of 5 min and 2 km near its final longitude, respectively, and extending to 5 km in the western United States. We downloaded AOD data and averaged the available values for each hour. The hourly data are assigned to the nearest cell of the considered 1 km grid. The different approaches to mapping coarser resolution 2–5 km ABI AOD pixels could lead to varying biases in the final grid. By utilizing the nearest-to-center 1 km grid cell simplifies the integration of the ABI AOD data into the overall analysis framework. Validating satellite remote sensing products is a crucial task in both their development and utilization. Zhang et al., Zhang and Kondragunta, Fu et al., and O’Dell et al. showed that GOES-R satellite potentially offer greater advantages in terms of spatiotemporal resolution for retrieving AOD compared to polar orbiting satellite.^30,32−34^

#### 2.1.4 Meteorological Variables

HRRR data, available at https://rapidrefresh.noaa.gov/hrrr/, is an atmospheric model in real time operation managed by the NOAA National Centers for Environmental Prediction.^35,36^ This model incorporates radar data every 15 min within a 1-h time frame to enhance the level of detail beyond what is offered by its predecessor’s (Rapid Refresh model) output on hourly basis. It has demonstrated accuracy in estimating observations of air temperature and dew-point temperature close to the surface.^37^ Moreover, the model was employed to assess wind, temperature, and humidity conditions close to the surface during wildfire events. When integrated with GOES data, it has proven advantageous in enhancing model efficiency for the calculation of geophysical processes, including real evapotranspiration.^38^ For the current study, we aggregated acquired 15 min meteorology parameters to hourly data, encompassing 2 m temperature, u- and v-wind components, planetary boundary layer height, shortwave and longwave flux, specific humidity, surface pressure, and relative humidity. The hourly data was obtained at 3 km from HRRR. The data were downscaled to a 1 km scale using inverse distance weighted (IDW) interpolation.

#### 2.1.5 Ancillary Variables

Goddard Earth Observing System Composition (GEOS-CF) was used to obtain AOD data for California (https://gmao.gsfc.nasa.gov/weather_prediction/GEOS-CF/). The GEOS-CF meteorological data was assimilated from a variety of conventional and satellite-driven data sources. Keller et al. described the detailed assimilation approaches.^39^ The specific AOD fields we used are to encompass optical depths for black carbon (BC), organic carbon (OC), nitrate, dust, sea salt, and sulfate. The GEOS-CF data have a native spatial resolution of 0.25°, and we further adopted a bilinear interpolation approach to align with our model resolution.^40,41^ Below is the formula for bilinear interpolation. Please refer to the descriptions below.
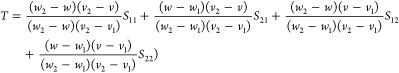
1where T is the target point. S_11_, S_12_, S_21_, and S_22_ are the bottom-left (w_1_, v_1_), top-left (w_1_, v_2_), bottom-right (w_2_, v_1_), and top-left (w_2_, v_2_) points, respectively. w and v are the coordinates for target point R. GEOS-CF hourly AOD interpolated to a spatial resolution of 1 km is merged with 1 km aligned HRRR hourly data. Subsequently, a connection between the GEOS-HRRR data and the GOES AOD was established through a nearest neighbor match, ensuring that each GOES AOD is mapped to a unique 1 km grid cell, thereby avoiding duplications.

### 2.2 Wavelet Transform

Wavelet signal processing can represent signals with sparse characteristics, capture the ephemeral features, and facilitate multiresolution analysis. The application of wavelets has been observed across various fields, such as remote sensing, medical imaging, geophysics, and computer vision.^42−44^ By decomposing signals or images into separate frequency components at various spatial scales, one can examine the underlying structures and patterns inherent in the data, gaining valuable insights. Wavelet analysis entails expressing functions as the sum of base functions. It encompasses the segmentation of a function or signal into distinct components, incorporating individual undulations (i.e., wavelets) on various scales. This enables the identification of distinct or localized fluctuations in the morphology. Transient features, often generated by impulsive system actions, often suggest a cause-and-effect relationship with an event. For instance, heartbeats produce peaks in an electrocardiogram signal. For PM_2.5_ prediction, the independent impact of high and low frequencies of meteorological parameters obtained using the Discrete Wavelet Transform (DWT) over the spatial domain will be reflected in the model. Sudden occurrences such as wildfires and the resulting spikes in air pollution, characterized by their noncontinuous and brief nature, can be detected using this method. Short-term fluctuations might indicate the movement of a weather system, and DWT enables the extraction of such relevant features, therefore allowing a PM_2.5_ prediction model to learn how these features may be correlated with PM_2.5_ concentration levels.

DWT comprises signal breakdown (decomposition) and reconstruction. It is a procedure of breaking down the signal into low-frequency (approximate) and high-frequency (detail) components using base wavelets. This process is typically carried out repetitively, gradually decreasing the frequency or increasing the scale. At a decomposition scale, approximation (l_J_) and details (h_J_) are derived. Hence, S (the original signal) at decomposition scale J is expressed as S = l_J_ + h_1_ + ... + h_J_. Within the framework of DWT, the essential requirements include the choice of a mother wavelet function and the decomposition scale. We tested several mother wavelet functions using the MATLAB wavelet toolbox for decomposing the input parameter and extracting low-high frequency components. We considered three wavelets: Haar, Daubechies, and Symlets. Following multiple iterations of testing, the db5 mother wavelet was chosen, and the decomposition scale was optimized.

The input signal encompasses diverse frequency components, wherein the contributions of low and high frequency components to the dynamic characteristics of the data vary across the domain.^45^ Upon decomposing the input variables using wavelet transform, significant components were identified through the feature selection algorithm. In the present study, we first trained the input data using a random forest. The input parameters which were less important based on the random forest variable importance were decomposed to extract significant components, whose relationships with PM_2.5_ were then learned by the random forest model. Another reason to decompose was the high variability observed in parameters like AOD. AOD was decomposed to train the model using low and high frequency components, aiming to capture the peaks and improve accuracy in predicting high levels of PM_2.5_. Using the wmaxlev function, we obtained the maximum decomposition level of 15 for the present data set. Level 5 was chosen as the optimal level of decomposition as the detail at this level becomes smooth enough to capture high frequencies. Yang et al. investigated the determination of the decomposition level, concluding that the selection should be guided by the characteristics of the analyzed series rather than being influenced by factors such as series length.^46^ Their study emphasized that inappropriate decomposition of the original series could adversely impact the performance of wavelet-aided modeling. We verified the choice of decomposition level for the present study along these lines, and our model showed best accuracy at level 5.

### 2.3 Model Development

We integrated AOD gap filling, wavelet transform, and random forest algorithm to predict PM_2.5_ levels. The random forest algorithm can be conceptualized as an ensemble regression technique that employs multiple decision trees.^47^ During training, the data set utilizes the bootstrap resampling method to build m decision trees, each based on a different sample from the data set. The dependent variable is derived by averaging the predictions generated by these decision trees. Random forest builds numerous decision trees to capture PM_2.5_ and its predictors nonlinear associations. The final output is determined by averaging predictions of PM_2.5_ from the individual trees. This technique not only offers rapid learning and prediction but also mitigates overfitting risks due to its randomized sampling strategy. Although various random forest-based models have been proposed to estimate PM_2.5_ levels,^15,28,48^ the insights into the relative significance and contributions of various predictors offered by this algorithm are important for our demonstration of the wavelet transform technique.

**Figure 2:**
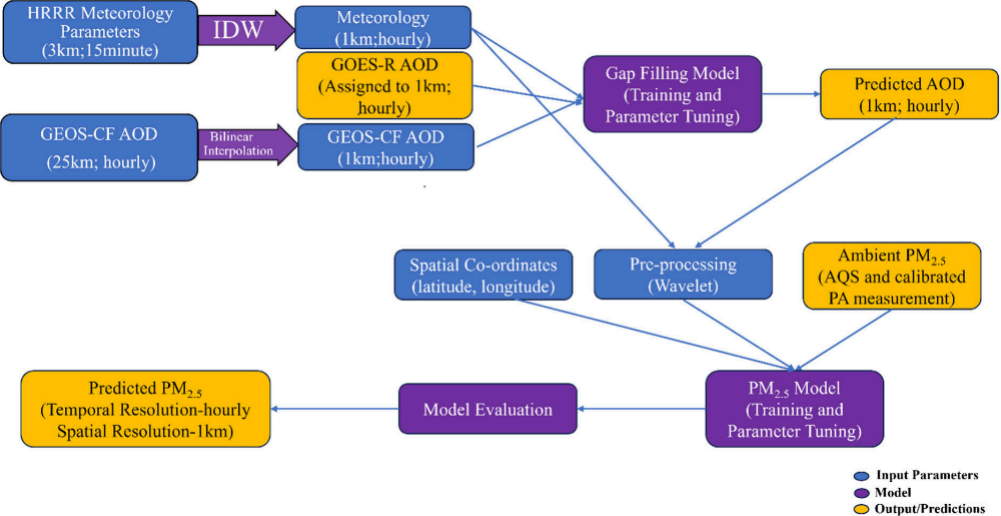
Workflow for PM_2.5_ prediction.

The PM_2.5_ prediction algorithm is illustrated in the flowchart depicted in Figure 2. First, hourly GOES-R AOD data were allocated to the nearest 1 km grid cell. Hourly total AOD from GEOS-CF was scaled to a resolution of 1 km. The scaled meteorological parameters at 1 km and hourly temporal resolution (as mentioned in section 2.1.4) along with GEOS-CF AOD were used as predictors for GOES-R AOD gap filling in a random forest model. A separate gap-filling model was trained every hour. Our approach ensures a comprehensive and accurate representation of AOD, incorporating both satellite measurements and additional atmospheric parameters for enhanced prediction and spatial coverage. We utilized the random forest package of R (R Development Core Team, 2008) for the prediction.^49^ We determined hyperparameter values that minimized the OOB error for the current hour, specifically setting ntree and mtry to 500 and 4, respectively.

Second, considering the high prediction accuracy achieved by the AOD gap-filling model, the hourly predicted AOD at 1 km resolution was employed as a predictor to estimate ground-level PM_2.5_. In our analysis, the dependent variable is the hourly ground-level PM_2.5_ observations, and the predictors comprise the gap-filled AOD for the same hour, meteorological variables, and spatial parameters (latitude and longitude). We processed AOD, longwave flux, u-wind, surface pressure, planetary boundary layer height, and upward shortwave radiation using the basis function of the Daubechies wavelet of order 5 (db5). For each original parameter (e.g., AOD, longwave flux, u-wind, etc.), this approach generated six wavelet components to be included in the random forest model, i.e., one approximation coefficient capturing the low frequency/smooth trend of the signal,and five detail coefficients capturing five high frequency/more rapidly changing patterns of the signal. The significant wavelet components, including AOD l_5_ (low frequency) and h_1–5_ (high frequency; level 1 to 5), are considered as inputs of the final PM_2.5_ prediction model. For specific humidity, longwave flux, u-wind, surface pressure, planetary boundary layer height, only the low-frequency component l_5_ was included based on their significance. In the case of upward shortwave flux, h_1_, h_3_, and l_5_ were considered as predictors due to their high significance. After the wavelet decomposition process, predictors with near-zero significance values were omitted from the final model. These wavelet components, combined with 2 m temperature, v-wind components, shortwave flux, relative humidity, and spatial parameters, were utilized to train the final random forest model.

We evaluated the performance of our hourly PM_2.5_ prediction model by comparing predicted values with ground observations in the context of 10-fold cross-validation (CV). In this approach, the training set was randomly and equally divided into 10 subsets. The model was run 10 times, with 10% of the samples left for testing each time, while the remaining 90% were used for training. CV R^2^ and root-mean-square error (RMSE) were calculated by comparing PM_2.5_ estimates with observations across the entire data set composed of the 10 test subsets. This process provided an estimate of the model’s predictive performance on unseen data. Moreover, we conducted 10-fold spatial CV to assess the accuracy of the model in arears without ground sensors. In spatial CV, the monitors were randomly divided into 10 rough hourly equal-sized groups. One group served as the test set, where PM_2.5_ measurements were excluded from the training process. The nine remaining groups constituted the training set. This process was carried out 10 times, once for each group, and the same validation metrics were applied to spatial CV. This approach evaluated the model’s ability to generalize to unmonitored locations, simulating real-world scenarios where PM_2.5_ predictions are required in areas lacking ground monitoring stations. Due to the drawback that EPA monitors are not uniformly distributed, we could not perform spatial clustering cross-validation. In some clusters, especially those with fewer monitors, they will have sparse data. On the other hand, a spatial buffer near Los Angeles and San Fransico will have majority of the ground sensors data.

## 3 Results

### 3.1 Overall Model Performance

The hourly gap-filled model for AOD achieved an average OOB R^2^ of 0.92 and an OOB RMSE of 0.05. We initially trained the PM_2.5_ prediction model with only AQS measurements as output, OOB R^2^ and RMSE observed were 0.75 and 14.69 μg/m^3^, respectively. The 10-fold spatial CV resulted in R^2^ of 0.54 and RMSE of 19.69 μg/m^3^. In the next step, we introduced calibrated PA PM_2.5_ measurements along with AQS measurements as output variables. OOB R^2^ increased to 0.84, and RMSE decreased to 10.46 μg/m^3^. Also, 10-fold spatial CV resulted in increase in R^2^ to 0.72, and RMSE decreased to 12.77 μg/m^3^. This improvement illustrates the significance of incorporating low-cost sensor measurements in enhancing model performance. The final model based on wavelet demonstrated an OOB R^2^ of 0.86 and RMSE of 9.27 μg/m^3^ (as shown in Figure S1). Additionally, the 10-fold spatial CV resulted in an R^2^ of 0.82 and an RMSE of 9.82 μg/m^3^ (Table S1 summarizes error statistics). Figure S2 represents the variable importance plot, which depicts less dependency on spatial parameters and more on geophysical predictors on using frequency components (wavelet). Despite successfully obtaining fine-resolution measurements with good accuracy, a crucial aspect of our model’s motivation was to assess its capability in predicting wildfire elevated PM_2.5_ levels. To evaluate the model’s performance in this context, we considered major wildfire events in California that occurred during the years 2020 and 2021.

### 3.2 Case Studies

#### 3.2.1 Case 1: 2020 Major Wildfires

In 2020, the August Complex and SCU Lightning Complex, both ignited by lightning on August 16, 2020, had substantial impacts on air quality. The August Complex spread across seven counties, Mendocino, Humboldt, Trinity, Tehama, Glenn, Lake, and Colusa—covering a vast area of 1,032,648 acres. This fire remained active for 87 days and was finally contained on November 11, 2020. In contrast, the SCU Lightning Complex encompassed 396,624 acres, affecting Santa Clara, San Joaquin, Contra Costa, Alameda, and Stanislaus counties, with containment achieved on October 1, 2020.

**Figure 3:**
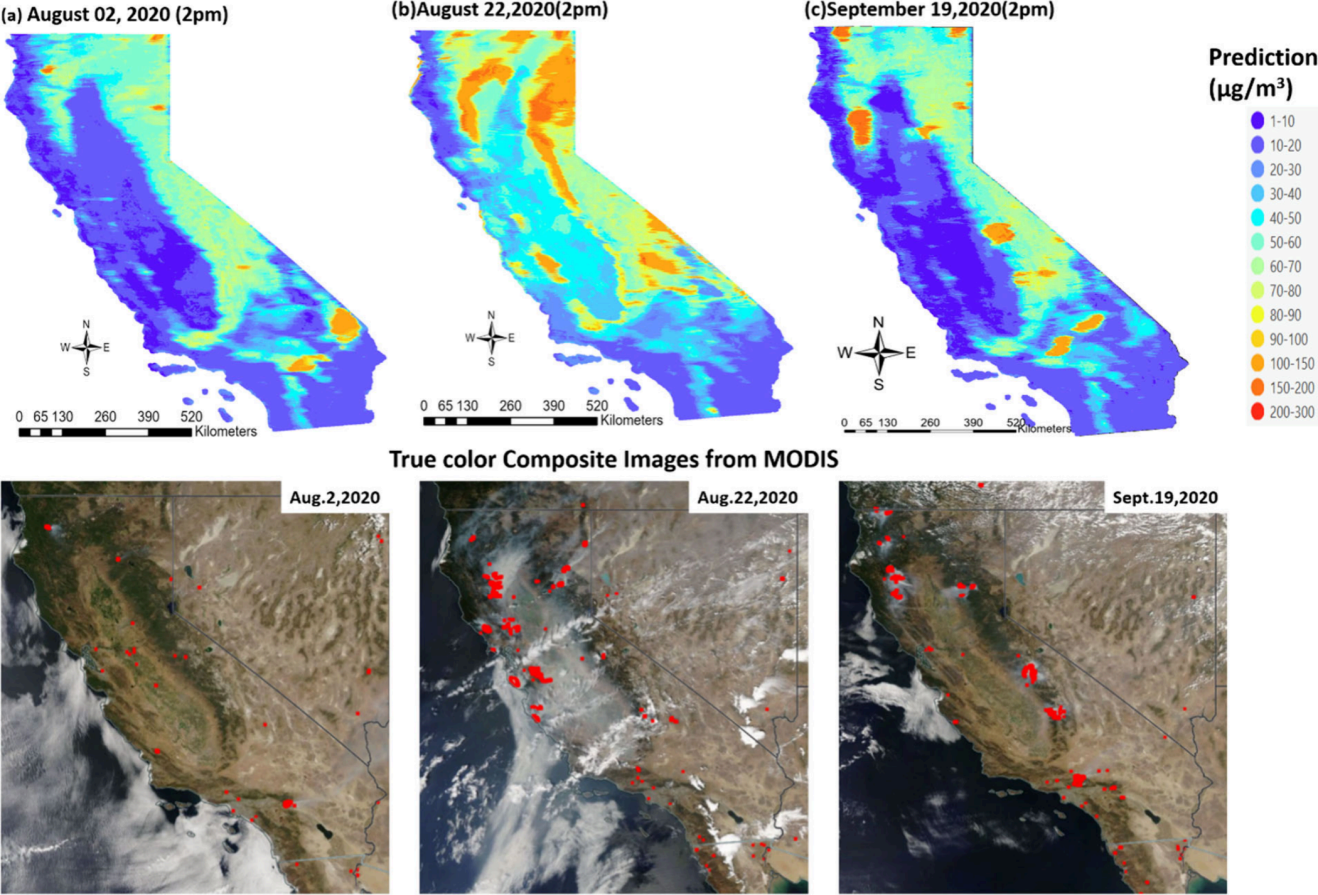
Comparison of hourly prediction map of PM_2.5_ in μg/m^3^ across California on (a) August 02, 2020, (b) August 22, 2020, and (c) September 19, 2020, with respective true color composite image from Aqua MODIS with active fire as red dots (Source: https://firms.modaps.eosdis.nasa.gov/).

On the following day of the above two major fires, August 17, 2020, the LNU Lightning Complex began its destructive path. Active for 46 days, it covered 363,220 acres and impacted five counties, Napa, Solano, Lake, Sonoma, and Yolo. All three major fires were active from August 17, 2020, to October 1, 2020. The following hourly prediction maps (Figure 3) illustrate ground-level PM_2.5_ measurements on selected days within this time frame, compared with true color composite images from Aqua MODIS. Aqua MODIS passes south to north over the equator in the afternoon.

**Figure 4:**
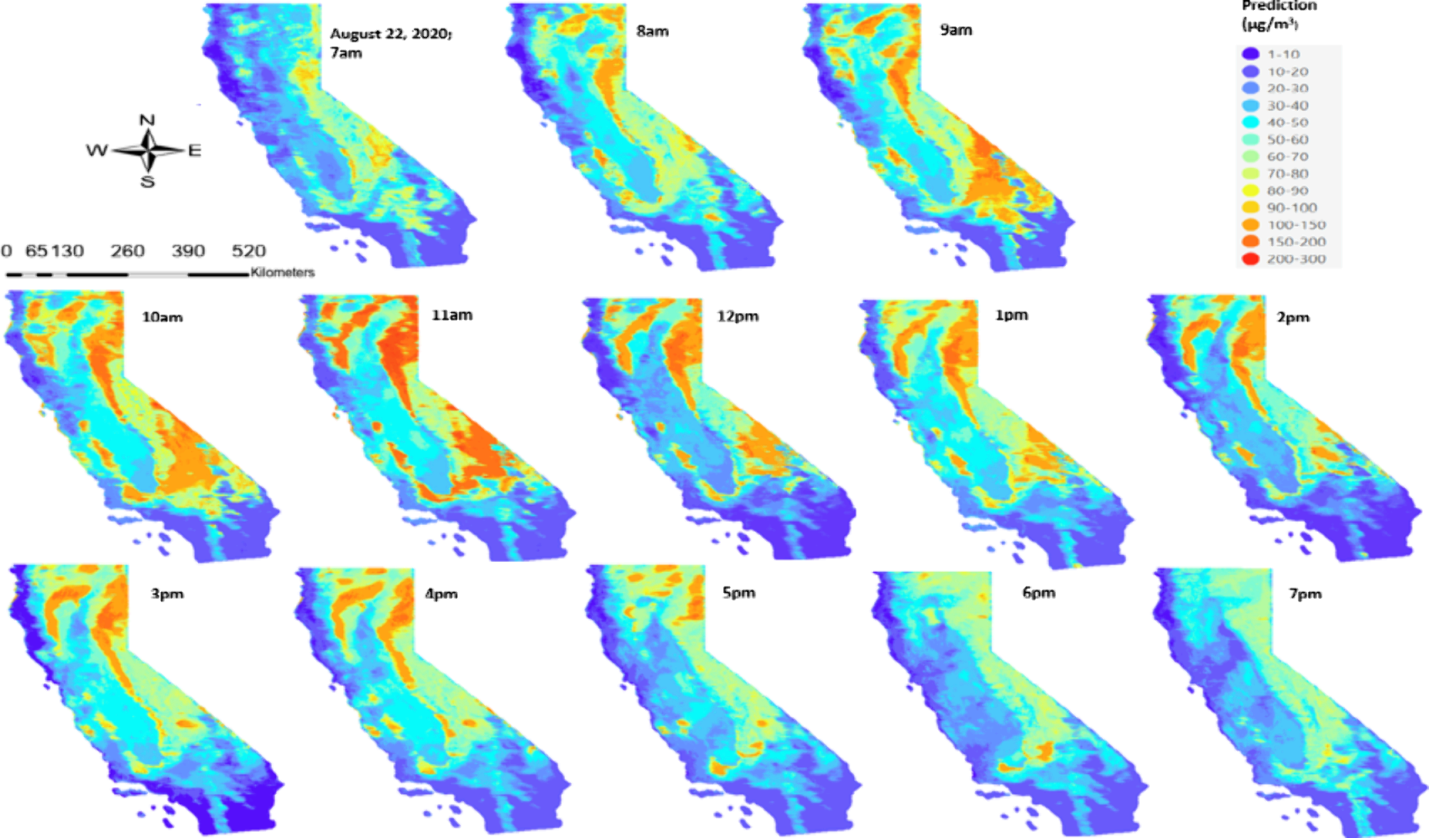
Hourly prediction maps of PM_2.5_(μg/m^3^) in California on August 22, 2020.

A comparison between our hourly PM_2.5_ predictions and true color composite images reveals strikingly similar smoke plume patterns in terms of spatial extent, suggesting a strong model performance. The prediction maps not only capture the major smoke plumes but also detect smaller fire episodes, as indicated by satellite-detected active fire spots, primarily in Southern California. The model demonstrates the capability of discerning spatial variability in PM_2.5_ levels across the domain. To delve deeper into the temporal aspects of our predictions, we plotted hourly time series prediction surfaces for August 22, 2020, showcasing significant variations in PM_2.5_ levels across different hours and spatial locations. Figure 4 illustrates the dynamic changes in the PM_2.5_ prediction maps between 7 am and 7 pm, capturing the evolution of smoke plumes and ground-level PM_2.5_ concentrations over time. Our hourly gap-filled satellite AOD-based PM_2.5_ predictions showed that a large amount of PM_2.5_ was produced starting from the early morning hours until noon, with noticeable decreases in concentration levels observed after 1:00 p.m. in the region. PM_2.5_ levels decreased from the 200–300 μg/m^3^ range in the afternoon to the 80–100 μg/m^3^ range during the evening hours. The high spatial (1 km) and temporal (hourly) resolution of the model enables detailed assessment of PM_2.5_ exposure patterns, which is crucial for evaluating potential health impacts and informing air quality management strategies, especially during high PM2.5 levels that can significantly degrade air quality over vast regions.

#### 3.2.2 Case 2: 2021 Major Wildfires

**Figure 5:**
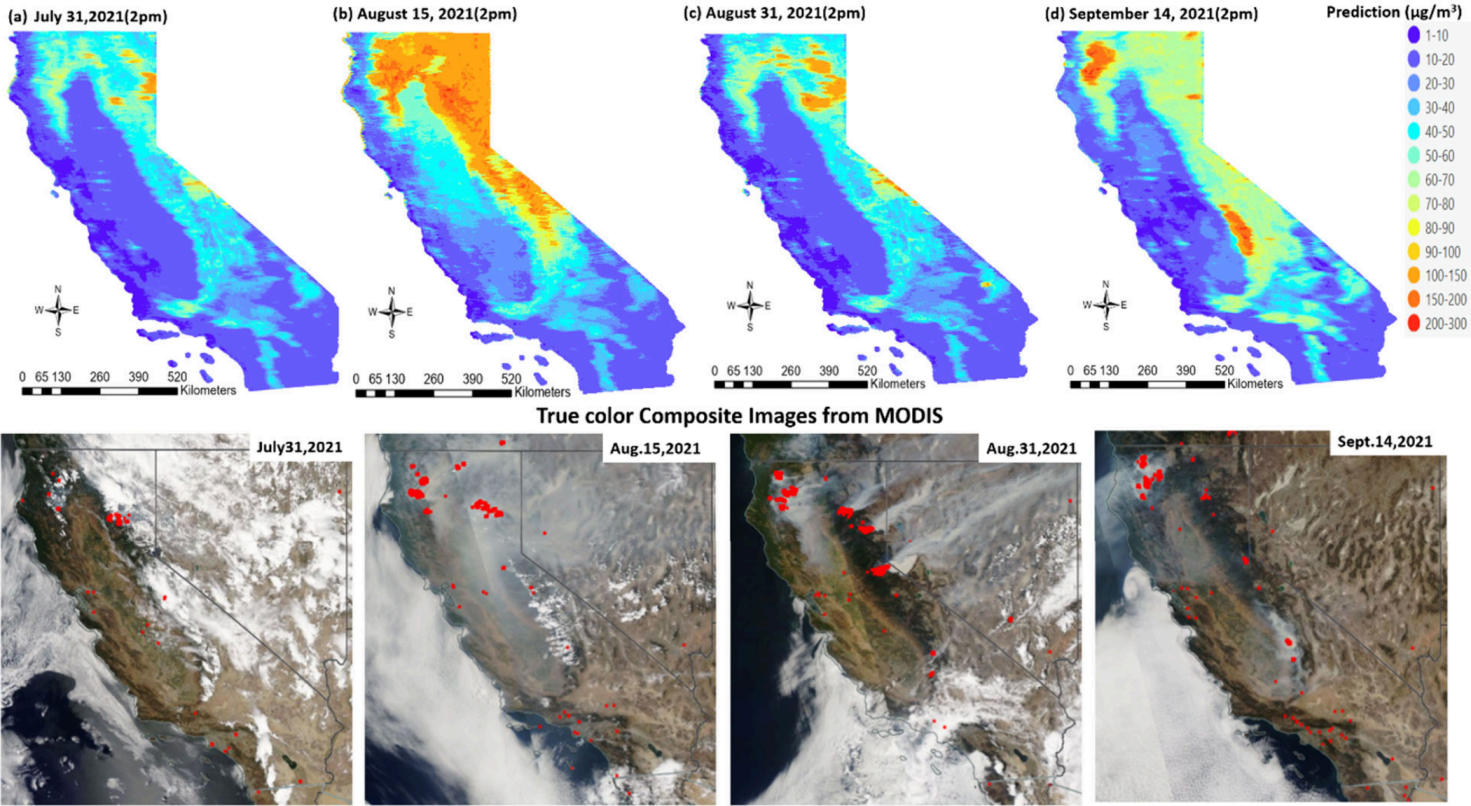
Comparison of hourly prediction map of PM_2.5_ in μg/m^3^ across California on (a) July 31, 2021, (b) August 15, 2021, (c) August 31, 2021, and (d) September 14, 2021 with respective true color composite image from Aqua MODIS with active fire as red dots (Source: https://firms.modaps.eosdis.nasa.gov/).

The significant wildfire events of 2021 included the Dixie, Monument, and River Complex Fires. The Dixie Fire, which ignited on July 13, 2021, spanned approximately 963,309 acres, achieved 94% containment after 104 active days, and led to the destruction of 1,329 structures. Its impact extended across five counties: Butte, Plumas, Shasta, Lassen, and Tehama. Both the Monument and River Complex Fires began on July 30, 2021, and were active for 88 days. The Monument Fire covered an area of 223,124 acres, while the River Complex Fire spanned 199,359 acres. Figure 5 shows hourly prediction maps within the time frame of July 30, 2021, to October 26, 2021. The spatial prediction plots show high PM_2.5_ concentrations due to the wildfires.

These high concentrations are depicted in the prediction maps, corresponding to the active fires observed in the true color composite images obtained from MODIS (Source: https://firms.modaps.eosdis.nasa.gov/). The initiation and cessation of wildfires are observable in Figure 5, from left to right. Concurrently, the prediction maps can be juxtaposed with the corresponding true color composite images from the MODIS, illustrating active fires and smoke plumes. The motivation behind section 3.2.1 and 3.2.2 is to compare smoke plume patterns with the spatial prediction plot. A comparison between hourly PM_2.5_ spatial predictions and true color composite images reveals strikingly similar smoke plume patterns in terms of the spatial extent. The major limitation of dealing with such events is reliable ground data, due to which deterministic statistics are difficult to evaluate.

## 4 Discussion

The association between PM_2.5_ and AOD has considerable spatiotemporal variability, posing a challenge for continuous and reliable PM_2.5_ prediction. Several previous studies have addressed this challenge by incorporating satellite data alongside convolutional layers of ground PM_2.5_ measurements as predictors.^13,14,37^ In our model, the low frequency component of AOD was the most important variable in explaining PM_2.5_ variability. When comparing error statistics, we considered the resolution and the model’s dependence on satellite AOD. The current study is very fine spatiotemporal resolution, and study area is California. Also, the main predictor is satellite AOD. This restricts us from comparing error statistics with just a few studies. Early studies using satellite AOD to estimate PM_2.5_ established the AOD-PM_2.5_ relationship using empirical statistical correlation,^50,51^ two stage generalized additive models,^52^ and land-use regression (LUR) plus Bayesian maximum entropy (BME)^53^ with CV R^2^ of approximately 0.60–0.81 at a coarse spatial resolution (8.9–12 km). Our proposed model took a different approach, aiming to unveil the spatiotemporal relationship between PM_2.5_ and satellite AOD using multiresolution analysis while achieving finer resolution in PM_2.5_ measurements.

Leveraging a combination of reanalysis AOD (offering seamless hourly data) and satellite AOD (providing high accuracy and spatial resolution), we present a novel approach to PM_2.5_ estimation. Without using reanalysis AOD, the observed values of the OOB R^2^ and RMSE were 0.59 and 12.57 μg/m^3^ using random forest. This new strategy demonstrated excellent performance in both cross-validation and mapping, ensuring no spatial discontinuity. The high spatiotemporal resolution PM_2.5_ predictions provided by our model contribute significantly to improving PM_2.5_ estimates, offering valuable insights for various health and environmental studies. Our research has significant implications for environmental monitoring and epidemiological assessments of short-term PM_2.5_ exposure. Satellite remote sensing offers extensive coverage of data for studying ambient air quality. Our study presents an effective method for harnessing the benefits of satellite AOD data as well as gridded meteorological fields in capturing spatiotemporal variations in ground PM_2.5_. The spatially resolved full-coverage hourly PM_2.5_ predictions can aid in evaluating the health effects of transient exposure to PM_2.5_ and guiding policy-making efforts for pollutant control.^54^

The findings of this study underscore the efficacy of wavelet transformation and machine learning as potent tools for capturing intricate spatiotemporal patterns that might be too nuanced for conventional statistical methods. Moreover, leveraging the GOES-R AOD product has substantial potential to supplement polar-orbiting satellite data and fill in data gaps, thereby enhancing the overall capabilities and insights derived from satellite observations. It is important to highlight a limitation of the present study that is the direction of our future work. Since AOD gap filling was done hourly, those hours without any successful AOD retrievals were not included in the following PM_2.5_ prediction model. As a result, while we possessed GOES-R AOD hourly data covering 86.5% of the total study period, we have not achieved complete temporal coverage to ensure precision in AOD measurements. Future work will consider moving the time windows to improve temporal data coverage.

Our high-resolution PM_2.5_ maps offer significant insights into the dispersion patterns of smoke plumes and the resultant impacts on air quality, thereby facilitating informed decision-making regarding public health advisories, emission mitigation strategies, and wildfire management initiatives. Furthermore, the model’s capability to capture rapid changes in PM_2.5_ levels highlights its potential utility in monitoring and estimating air quality during dynamic wildfire scenarios. Our approach proves to be versatile and can be applicable to diverse regions, covering areas with a mix of major urban centers, frequent smoke events, snow cover, and intricate terrains, where the traditional challenges of predicting PM_2.5_ are pronounced. States like California with substantial climate variability, complex topography, and frequent wildfires provide a good study domain to validate the model efficiency. This adaptability and consideration for uncertainty make our methodology valuable in addressing PM_2.5_ prediction challenges across a wide range of environmental contexts.

Our proposed method integrates GOES-R AOD products for fine-resolution predictions and employs wavelet transform techniques to mitigate random forest underestimation at elevated levels. This leads to an impartial and uninterrupted prediction of the PM_2.5_. It offers a novel way to preprocess input data, diverging from the utilization of intricate deep learning models that demand substantial computational resources. This not only serves as a valuable guide for refining our predictive models continuously but also plays a crucial role in the assessment of measurement errors in exposure and health studies.

## Supporting Information Available

Figure S1: OOB measured vs predicted PM_2.5_ (μg/m^3^) from SMOTE–random forest model and wavelet–SMOTE–random forest model. Figure S2: Variable importance plot: Random forest with SMOTE and wavelet random forest with SMOTE. Table S1: Error statistics. (PDF)
