# Supplementary material for: Multiresolution Analysis of HRRR Meteorological Parameters and GOES-R AOD for Hourly PM2.5 Prediction
Source: Environ Sci Technol. 2024 Nov 1;58(45):20040–8. doi: 10.1021/acs.est.4c03795 (PMC11562723; doi:10.1021/acs.est.4c03795)
Supplement: Supplementary file 1 — es4c03795_si_001.pdf [file es4c03795_si_001.pdf]

Supporting Information

Multi-resolution analysis of HRRR meteorological parameters and GOES-R AOD for hourly $PM_{2.5}$ prediction

Dimple Pruthi, Qingyang Zhu, Wenhao Wang, Yang Liu*

Gangarosa Department of Environmental Health, Rollins School of Public Health, Emory University, Atlanta, GA 30322, United States.

*yang.liu@emory.edu

Summary: 2 Pages, 2 SI figures and 1 SI Table

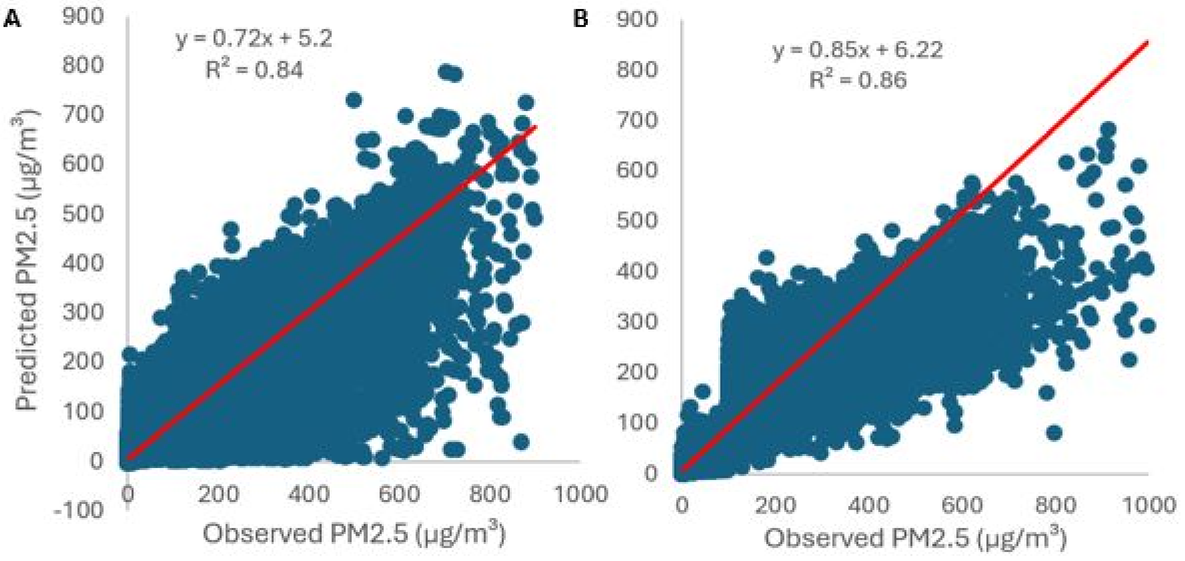

Supplementary Figure S1: OOB measured vs. predicted PM2.5(μg/m^3) from (A) SMOTE-Random Forest model (B) Wavelet-SMOTE-Random Forest model

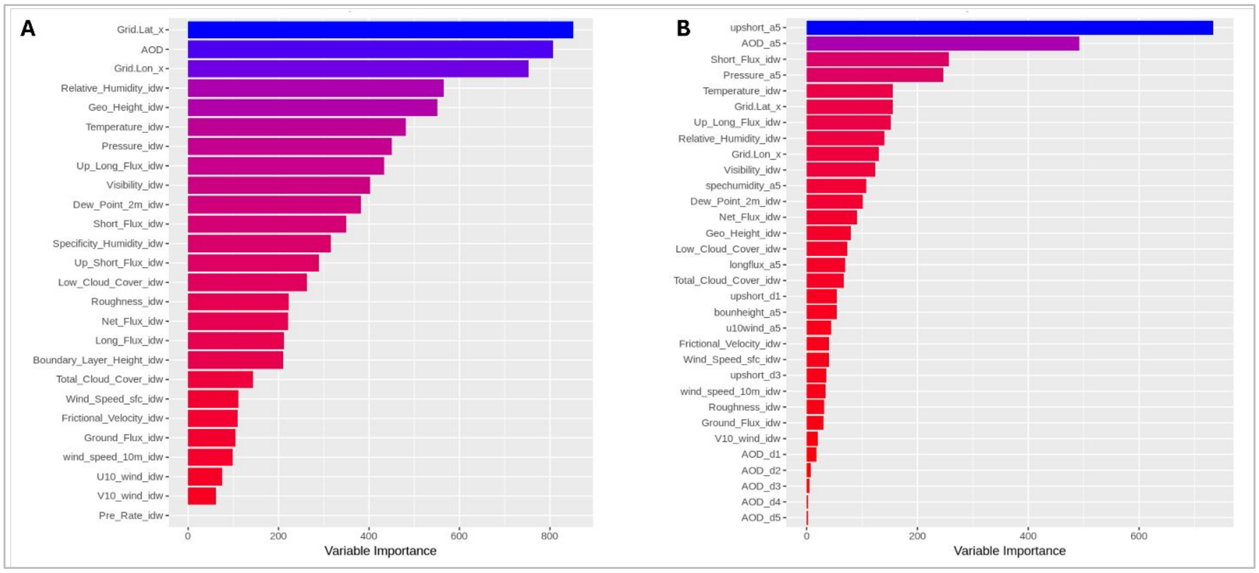

Supplementary Figure S2: Variable importance plot: (A) Random forest with SMOTE, (B) Wavelet random forest with SMOTE. (Up_Long_Flux – Upward Longwave Flux; Up_Short_Flux – Upward Shortwave Flux; Spechumid – Specific humidity; idw – Inverse Distance Weighted; di – Details at level i; ai – Approximation at level i; bounheight - planetary boundary layer height)

The variable importance describes the relative importance of each input feature. We can measure how each feature decreases the impurity of the split (the feature with highest decrease is selected

for internal node). For each feature we can collect how on average it decreases the impurity. The average over all trees in the forest is the measure of the variable importance.

Supplementary Table S1: Error Statistics

**Model (Target Variable)**	**Out-of-bag**		**Spatial 10-fold Cross Validation**	
	**R^2**	**RMSE (μg/m^3)**	**R^2**	**RMSE (μg/m^3)**
Random Forest (RF) (AQS PM2.5)	0.75	14.64	0.54	19.69
SMOTE-RF (AQS and PA PM2.5)	0.84	10.46	0.72	12.77
Wavelet-SMOTE-RF (AQS and PA PM2.5)	0.86	9.27	0.82	9.82
